# Paleoradiological and scientific investigations of the screaming woman mummy from the area beneath Senmut’s (1479–1458 BC) Theban tomb (TT71)

**DOI:** 10.3389/fmed.2024.1406225

**Published:** 2024-08-02

**Authors:** Sahar N. Saleem, Samia El-Merghani

**Affiliations:** ^1^Department of Radiology, Kasr Al Ainy Faculty of Medicine, Cairo University, Cairo, Egypt; ^2^Ministry of Tourism and Antiquities of Egypt, Cairo, Egypt

**Keywords:** ancient Egypt, screaming mummy, Senmut, computed tomography (CT), Wig, Fourier transform infrared spectroscopy (FTIR), Theban tomb 71 (TT71), Hatschepsut

## Abstract

**Introduction:**

The Screaming Mummy of Cairo Egyptian-Museum Store, is an anonymous woman with a wide-open mouth coded as CIT8, discovered beneath Theban Tomb 71 (TT71) which is the burial site of Senmut’s relatives, the architect of 18th-Dynasty Queen Hatschepsut (1479–1458 BC). The study aims to evaluate if combining computed tomography (CT) with scientific investigations and archeological data of the Screaming Mummy CIT8 will reveal information about its physical appearance, health, cause of death, and mummification.

**Methods:**

We CT-scanned the mummy and created reconstructed images. Scanning-Electron-Microscope (SEM), Fourier-Transform-Infrared-Spectroscopy (FTIR), and X-ray-Diffraction-Analysis (XRD) were used to investigate mummy skin, hair, and wig samples. We compared our findings to previous data.

**Results:**

Computed tomography estimated the age of death to be 48.1 years ±14.6 based on the pubic symphyseal surface. CT detected mild-to-moderate teeth attrition, and joints degeneration. The desiccated brain and viscera remained *in situ*. FTIR revealed the wig is formed of midrib date palm that shows in CT as spiral low density fibers. The wig fibers are partially coated with a thick substance that is inspected as black consolidation and identified as crystalline by XRD, comparable to material found in an ancient wig-making workshop. FTIR showed that the skin, hair, and wig samples were treated with imported juniper resin had anti-bacterial and insecticidal properties. The skin and wig samples contained frankincense, and the hair sample contained henna.

**Discussion:**

Combining the advantages of paleoradiology to the scientific investigations, provided enhanced comprehension of the mummy CIT8 and ancient Egyptian wig structure and material. CT scanning non-invasively showed the mummy’s inner and exterior morphology, and estimated the age of death as 48 years. CT evaluated the mummification technique based on retained viscera and absence of embalming packs. The scientific tests revealed expensive imported embalming materials, contradicting the traditional belief that the non-removal of the viscera implied poor mummification, resulting in careless embalmers sealing the mouth. The widely opened mouth could be a result of facial expression of suffering before death, fixed by cadaveric spasm. The study also explores how rigor mortis, tissue decomposition, burial techniques, and postmortem alterations may contribute to a mummy’s screaming appearance.

## 1 Introduction

The massive excavations that took place in Egypt in the 19th and early 20th century unearthed thousands of mummies (1). In 1935–1936, the Metropolitan Museum of New York excavated the hillside beneath Senmut’s Theban service tomb number 71 (TT71) in Luxor (2). Senmut was the architect of the Temple of 18th Dynasty Queen Hatschepsut (1479–1458 BC); he held various other positions and was important throughout Hatschepsut’s reign (3). The expedition discovered beneath TT71 tomb, a burial chamber built by Senmut for his mother Hat-Nufer, as well as individual burials of Senmut’s dead relatives and were brought to Thebes from another cemetery (2, 3). In burial number II, an unnamed woman’s mummy with a widely opened mouth was discovered in a wooden coffin (4). At the time of this examination, the mummy was kept in Cairo Egyptian Museum under the code CIT8 at the store for retrieved mummies from Kasr Al Ainy Faculty of Medicine. The Cairo Egyptian Museum refers to this mummy as the “Screaming Woman Mummy of the Store of Kasr Al Ainy” (5).

Mummies are well-preserved ancient remains referred to as “time capsules” because they can provide information about ancient people (1, 6).

Paleoradiology is a non-invasive study of mummies and archeological artifacts through the use of radiographic techniques: X-ray and computed tomography (CT). CT has been used to examine ancient Egyptian mummies. CT provides insight into the mummy’s morphology and internal preservation condition, mummification style, health conditions, and detecting likely causes of death (6–10).

Archaeometry is the use of scientific methodologies in examining and interpreting ancient remains and artifacts with the goal of better understanding past cultures. Modern scientific investigations, such as Scanning Electron Microscope (SEM), Fourier Transform Infrared spectroscopy (FTIR), and X-ray Diffraction Analysis (XRD), are available techniques widely applied to ancient human mummies and can provide specific information about the nature of funerary objects and embalming materials (11). The non-destructive nature of FTIR and SEM made them useful for the identification and characterization of ancient biological tissues, such as skin and hair. Ancient Egyptian embalming materials’ crystalline nature and chemical composition could be characterized by using FTIR and XRD (10–12).

Occasionally ancient Egyptian mummies were discovered with their mouths widely opened. Two royal mummies from the Deir el-Bahari royal cache, have their mouths widely opened, as though they were screaming: Prince Pentawere (1184–1153 BC) of the 20th Dynasty and Princess Meritamun probably the sister of King Ahmose (1550–1525 BC) of the early 18th Dynasty (6). CT examination of the mummified bodies of Pentawere and Meritamun provided insights to understand the cause of their physical appearance, health issues, and preservation condition. In this study, we investigated an ancient Egyptian mummy CIT8 with a wide-open mouth, as if screaming, using CT combined with scientific analytical tests aimed at assessing if this combination would reveal pathological abnormalities, help in understanding the natural history of ancient diseases, detect likely causes of death, and explore mummification techniques.

## 2 Materials and methods

### 2.1 Material historical background

The Mummy investigated in this study, coded as CIT8, is stored at Cairo Egyptian Museum in the Store of the retrieved Mummies from Kasr Al Ainy Faculty of Medicine. According to Cairo Museum’s registration book, the mummy was discovered by the Metropolitan Museum’s excavations (season 1935–1936) below the Service Tomb of Senmut (TT71) in Deir el-Bahari, Luxor. The Mummy was given to the Kasr Al Ainy Faculty of Medicine, where it remained until 1998, when the Egyptian Museum in Cairo requested to have it removed to be stored at the Museum.

The Metropolitan Museum in New York City-United States provided the excavation notes of this mummy upon the authors’ request: Metropolitan Theban Cards number 4992-3 (4). According to these excavation notes, the mummy was labeled as (Anonymous XVIII Dynasty Burial No. II). The mummy was discovered intact inside a wooden anthropoid coffin that measured 195 cm in length, 54.5 cm in width, and 70 cm in depth. The coffin’s box and lid are crudely carved from two sycamore wood sections, and it is covered in painted gesso that features a blue and yellow wig cover, a wide collar, and body bands without any inscriptions. The coffin is currently exhibited at Gallery 116 of the Metropolitan Museum in New York under Accession Number 36.3.184.^1^

The excavation notes (4) included the detailed description of the examination of the mummy at the time of the excavations (1935–1936):

“Height length,” heels to crown of head: 158 cm.
Condition: A well-preserved mummy. Skin all present tanned hard and thick, and a deep reddish brown in color. White mold around legs and feet and over breast. Finger and toenails preserved. Eyes tight closed (lower lids flattened down). Vagina open, mouth wide-open.
Position: on back, fully extended; legs together; hands side by side, open, palms down, over the pudenda; head level (chin pulled down and in slightly).
Mummification: Thoroughly “cured” (skin tanned) in a (salt) solution and drenched in a dark oily liquid preservative. Neither brains nor viscera removed (nose closed still; no incision anywhere on body). Sex; Female. Age: Elderly adult.
Teeth: Large, white, and strong. Much worn. Missing (lost during lifetime): 2nd–3rd molars, upper right; third molar, upper left; 1st–2nd molars, lower right; second bicuspid, lower left.
Hair: (on crown of head) Thin, fairly long, slightly wavy; now pale yellowish brown in color probably white or gray at time of death. False hair: on either side of the head, braided to the woman’s own sparse hair at either side of the crown of the skull, a long heavy roll or switch, composed of fine braids of very dark brown human hair (tapering in slightly at each end, the lower ends whipped with loose strands of hair)—altogether similar to those on the head of the body of Hat-Nufer (Tomb of Ra′-Mose and Hat-Nufer burial No. II, body) and similarly applied; but with the lower ends of the switches hanging loose over the shoulders, instead of being bound together over the breast.

Objects on the body: on the third finger of the left hand of the mummy, two scarab rings:

- The inner ring on the finger red jasper scarab on a thin silver ring.
- Green jasper scarab, with gold swivel mounting and gold ring.

These two rings are currently displayed at the Metropolitan Museum of Arts in New York (Supplementary Figure 1).

**Figure 1:**
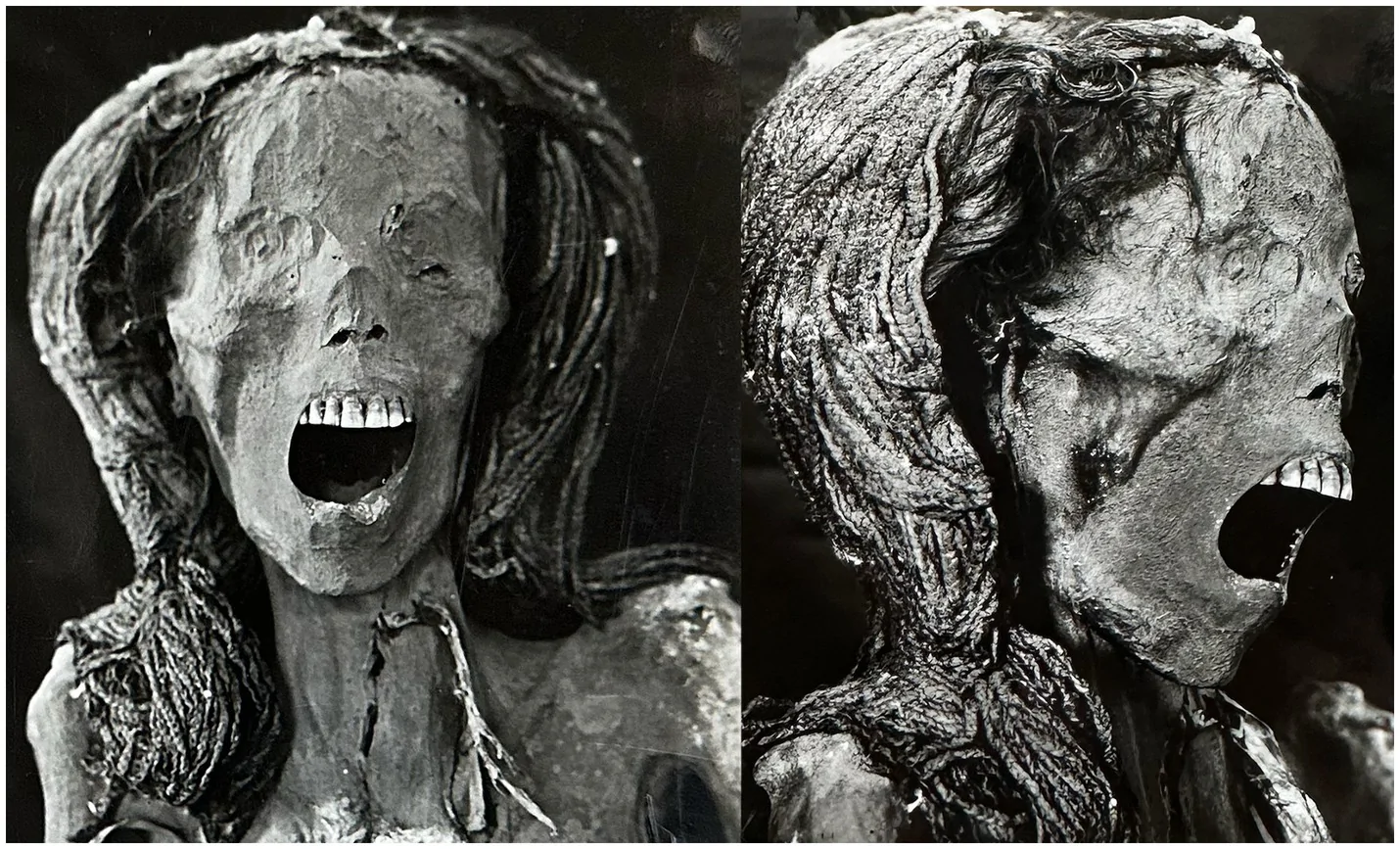
Photographs of the front and right profile of the head of an anonymous mummy (referred to as CIT8) were taken in 1939 at the Kasr Al Ainy Faculty of Medicine in Cairo, Egypt. The mummy was discovered in Deir El Bahari (Luxor, Egypt), near the service tomb of Senmut (the architect during Queen Hatschepsut’s reign 1479–1458 BC). The photographs show the mouth is widely opened as if screaming. The mummy wears a wig that is divided into two separate parts that extend down both sides to the level of the shoulders. The wig’s two sides are joined on the head. The wig is composed of dark long braids. The mummy’s natural hair is visible near the front of the right side of her head.

The mummy’s accessible historical photos were taken in 1998 at the Cairo Egyptian Museum for the full body, and at Kasr Al Ainy Faculty of Medicine in 1939 for the head and neck region (13) (Figure 1).

### 2.2 Methods

The authors were granted permission by the Egyptian Antiquities Permanent Committee to examine the mummy CIT8.

#### 2.2.1 Physical examination

We physically examined the mummy and noted its posture, physical appearance, and the wig it is wearing. We took photos of the mummy. We matched the results of the current inspection to information found in the excavation cards of the Metropolitan Museum from 1935 to 1936 and to images of the mummies that were taken at Kasr Al Ainy Faculty of Medicine in 1939, and upon its arrival at Cairo Egyptian Museum in 1998.

We scanned the mummy using computed tomography (CT) and created reconstructed images. Scanning Electron Microscopy (SEM), Fourier Transform Infrared Spectroscopy (FTIR), and X-ray Diffraction Analysis (XRD) were used to investigate mummy skin, hair, and wig samples. We compared our findings with previous data.

#### 2.2.2 CT scanning

We moved the mummy from the store located at Cairo Egyptian Museum to the CT scanner (Somatom Emotion 6; Siemens Medical Solutions, Florsheim, Germany) mounted on a truck at the Museum’s garden.

We employed the following CT parameters: Field of View (FOV): 320–500; slice thickness: 0.625–1.25 mm; reconstruction thickness: 0.4–0.8 mm; kV = 130; effective mAs: 23–63; pitch: (0.83–1.8).

Utilizing six slice CT scanner, the key imaging strategies that lead smooth imaging filters with 20 kernels for whole-body scans to improve soft tissue differentiations, and high-resolution imaging kernels (B60s, H70s, and U90s) for bone, dentition, and required body structures such as symphysis pubis. These techniques allow for greater visualization of anatomical details, which aids in precise internal structural evaluation and 3D modeling. To yield isotropic voxels, the whole mummified body was scanned in sections using 0.625 slice thickness and 320 mm FOV. To scan a larger FOV of 400–500 of the mummy’s body, we used a slice reconstruction thickness of 1.25 mm and B20 imaging kernels (Supplementary Table 1).

After generating axial images, we created two- and three-dimensional CT scans using specialized reconstruction software (OsiriX, Pixmeo SARL, Bernex, Switzerland). By using three-dimensional models, it was possible to virtually rotate the mummy in every direction without physically moving it.

We used previously published protocols to analyze the mummy’s CT images in order to estimate its age of death, identify pathologies, and state of preservation (6, 14–16). Epiphyseal union occurs along a growth algorithm, which can be used for estimating the age of death. The epiphyseal union status of long bones can be identified through coronal and sagittal reconstructed images (6). In youth, pubic symphyseal face morphology is undulating, but it smoothes out with age and can be estimated in phases using the Suchey-Brooks method (14). The CT virtual cut tool is used to cut the midline of the symphysis pubis and expose the symphyseal surface (14, 15). Multiplanar reconstructed CT scans can reveal age-related degenerative changes in joints, including the spine. The mummy’s height is measured from the top of the skull (vertex) to the heel using sagittal reconstructed CT scans. The living stature of the studied woman was estimated from tibial bone length measurements using Raxter’s regression-equations derived from Egyptian population. The tibia was preferred as it has lower standard error of the stature estimate than the femur (16).

We measured mummy’s stature, long bones, and structures (in millimeters) in CT images by marking the reference points and calculating the distance between them.

Using the region of interest (ROI) tool, we calculated the CT density of the mummy tissues and any foreign bodies in Hounsfield units (HU). We compared the CT results of the mummy with the relevant archeological information and literature.

For the objective assessment of visceral preservation, we utilized Aufderheide’s visceral organ preservation index (visceral index) for women mummy. There are 10 organs listed in the visceral index, including the heart, lungs, liver, spleen, kidneys, bladder, intestine, uterus-ovaries, breasts, and hair. Each organ is awarded 10 points if it is recognizable grossly, with total sum equaling the percentage of visceral preservation (visceral index) (17). Preserved soft tissues were searched for in their approximate anatomical positions in the multiplanar reconstructed CT images as relatively hyper-dense structures with different degrees of shrinkage or collapse.

#### 2.2.3 Investigations of mummy’s samples

We extracted samples that were needed for the study of the mummy’s skin, hair, and wig.

We removed two pieces of fragile dry skin samples measuring 12 mm × 10 mm and 10 mm ×11 mm from the mummy’s fractured front wall of the torso region. We took sample from the hair at the front of mummy’s head that measured (41 mm in length). We took a sample from a broken part of the wig hair that measured (50 mm × 10 mm).

We investigated the mummy’s samples using the following methods:

- We used Scanning Electron Microscope (SEM) (SEM QUNTA FEG 250**-**NRC) with different magnification of 200, 400, 800, 1,500, and 3,000 to study the natural hair and wig samples. SEM is used to obtain magnified images of samples in order to investigate their form and morphological structure.
- We used Fourier Transform Infrared spectroscopy (FTIR) (IR machine, Bruker, model Vertx 70 with ATR unit) to identify any material on the mummy samples of skin, natural hair, and wig. FTIR is a non-destructive analytical method that can be used to distinguish between polymeric, organic, and occasionally inorganic materials. Infrared light is used in the FTIR analysis procedure to scan test materials and observe chemical characteristics. The sample’s molecular makeup and structure are determined by measuring its capacity to absorb infrared light energy at different wavelengths (absorption curves) (11). We compared the characteristic absorption curve of the wig hair sample with different plant fibers usually used in manufacture of ancient wigs: including papyrus, flax, palm fibers coir, and date palm midrib.
- We used X-ray Diffraction Analysis (XRD) (Philips PW 1710 diffractometer, cupper tube anode) on part of the wig sample that measured 10 mm × 10 mm. XRD is an analytical technique using monochromatic *x*-ray allows verification of the crystallinity and structure of a material (11). We used the XRD analysis on the surface of the wig’s sample.

## 3 Results

### 3.1 Physical appearance of the mummy

**Figure 2:**
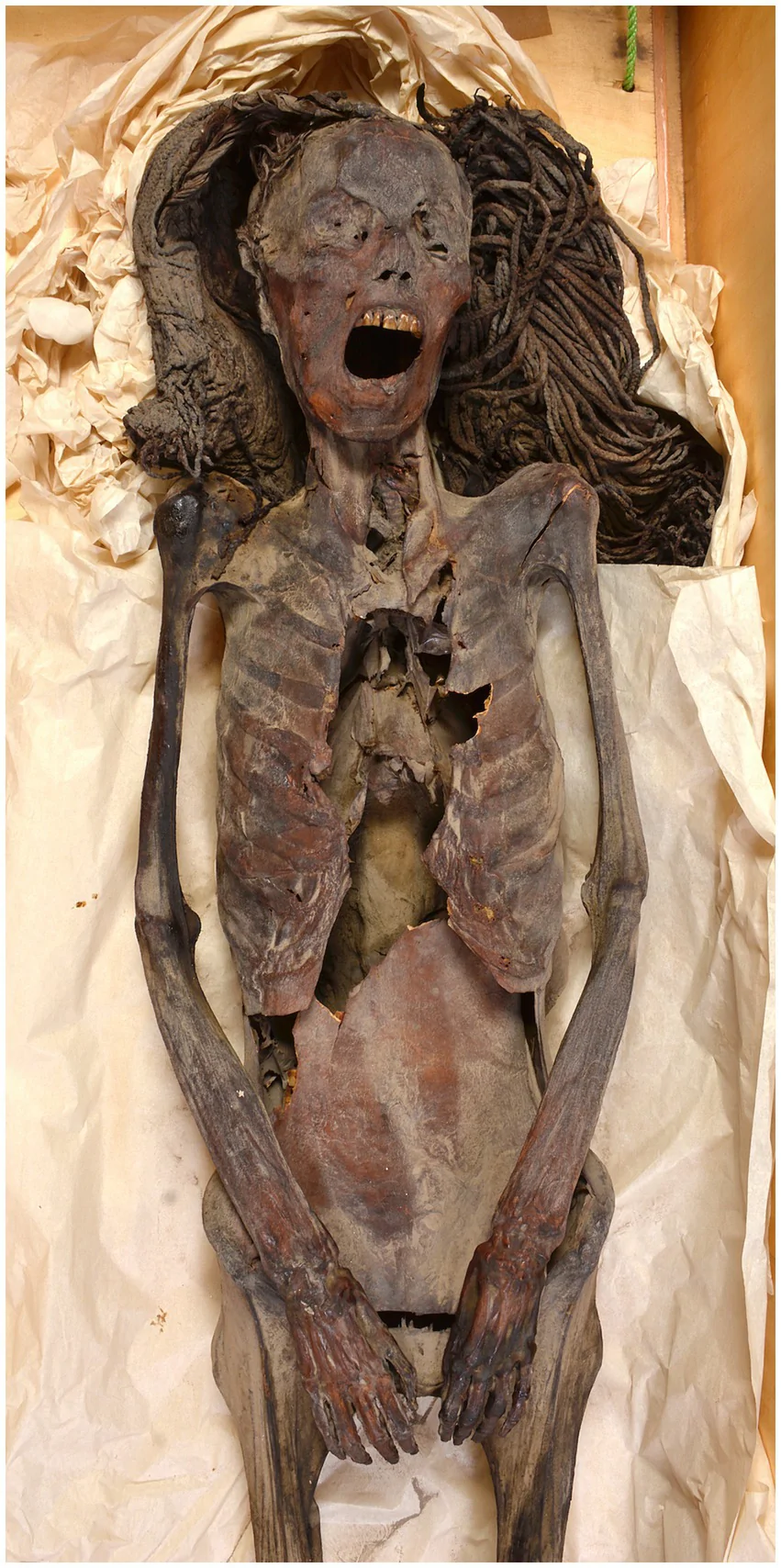
A photograph of the head to the midthighs of an anonymous mummy (referred to as CIT8) was taken in 2023 at the Egyptian Museum in Cairo, Egypt. The mummy CIT8 was discovered in Luxor, Egypt, near the service tomb of Senmut, the architect during Queen Hatschepsut’s reign 1479–1458 BC. The photograph shows the mummy in a supine position, arms extended and hands on the groin. The skin has a dark brown color. A rectangular defect with sharp, regular edges is seen in the center of the chest’s anterior wall. The anterior abdominal wall has broken and collapsed.

The mummified body was not wrapped which allowed visual inspection. The mummy is lying in a supine position with extended legs. The two arms are extended with the forearms angle inward so the hands with extended fingers are positioned on the groin (Figure 2).

The mummy is in good condition. The mummified body’s skin appears dark brown in color. At the center of the anterior wall of the chest, there is a rectangular defect with sharp, regular margins. The anterior abdominal wall is fractured and collapsed. There is no embalming incision. The perineal openings (vagina and anus) are rounded and noticeably enlarged.

**Figure 3:**
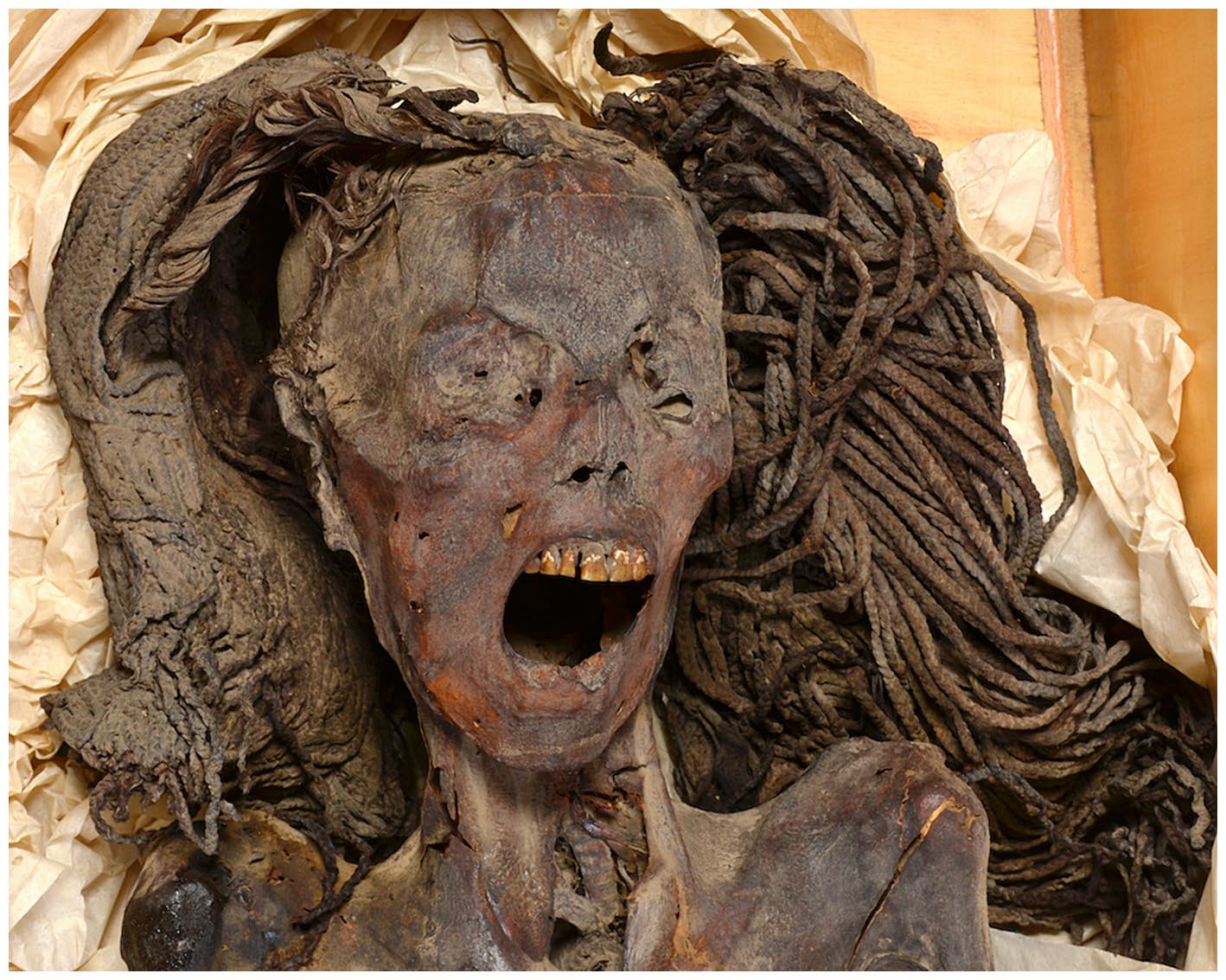
A photograph of the front of the head of mummified woman CIT8 was taken in 2023 at the Egyptian Museum (Cairo, Egypt). The mummy was discovered in Luxor, Egypt, near the service tomb of Senmut, the architect during Queen Hatschepsut’s reign 1479–1458 BC. The mummy’s mouth is wide-open, as if screaming. The mummy wears a wig consisting of braids some inspected with black consolidated material on. The wig is formed of two sections that extend down both sides to the level of the shoulders. The wig sections are disconnected and widely spaced at the top of the head.

The head is slightly tilted to the left side and the chin pulled down slightly. The eyes are closed. The mouth is widely opened. The teeth are worn with multiple missing teeth including: upper right third molar tooth, upper left third molar; second and third lower right molars, and lower left second premolar tooth. Partially broken teeth: right upper lateral incisor and right lower first molar tooth. Short scantly tuft hair appears at the head front. The mummy wears a wig that is divided into two sections on either side of the head, braided to the woman’s own sparse hair. The two parts of the wig are widely separated on the top of the head and extends down on both sides to the level of the shoulders. The wig is made up of dark brown long braids with twisted ends and is partly topped with a black consolidated material (Figure 3).

In comparison to the photographs of the mummy obtained in 1998 upon arrival of the mummy to Cairo Egyptian Museum from Kasr Al Ainy Faculty of medicine, the defect at the central part of the anterior chest wall is seen while the anterior abdominal wall was intact and not collapsed. In mummy’s head photos taken in 1939, the wig’s two sides were still connected on the head.

### 3.2 CT study of the mummy

#### 3.2.1 Preservation status

A large rectangular defect in the central part of the anterior wall of the chest measures 248 mm × 99 mm in craniocaudal and transverse dimensions, respectively. The defect involves the currently missing lower manubrium, body, xyphoid as well as the medial ends of the attached ribs on both sides. The defect’s margins are sharp regular and show no evidence of bone healing. The body cavity beneath the defect was examined, but no signs of missing bone fragments were found. The lower anterior abdominal wall has lateral cracks and collapsed into the body cavity. Missing of the distal phalanx of the right first and fifth toes. Otherwise, intact articulated skeleton with overlying intact thin desiccated soft tissues.

Seven of the 10 visceral organs listed in Aufderheide’s visceral index were identified in CT images of mummy CIT8. In torso CT images, the heart, lungs, liver, spleen, kidneys, and intestine were observed as desiccated, relatively hyperdense structures in their approximate anatomical positions. Locks of natural hair were observed on the side of the head, yielding a total visceral index of 70% suggesting good visceral preservation.

#### 3.2.2 Age at death

**Figure 4:**
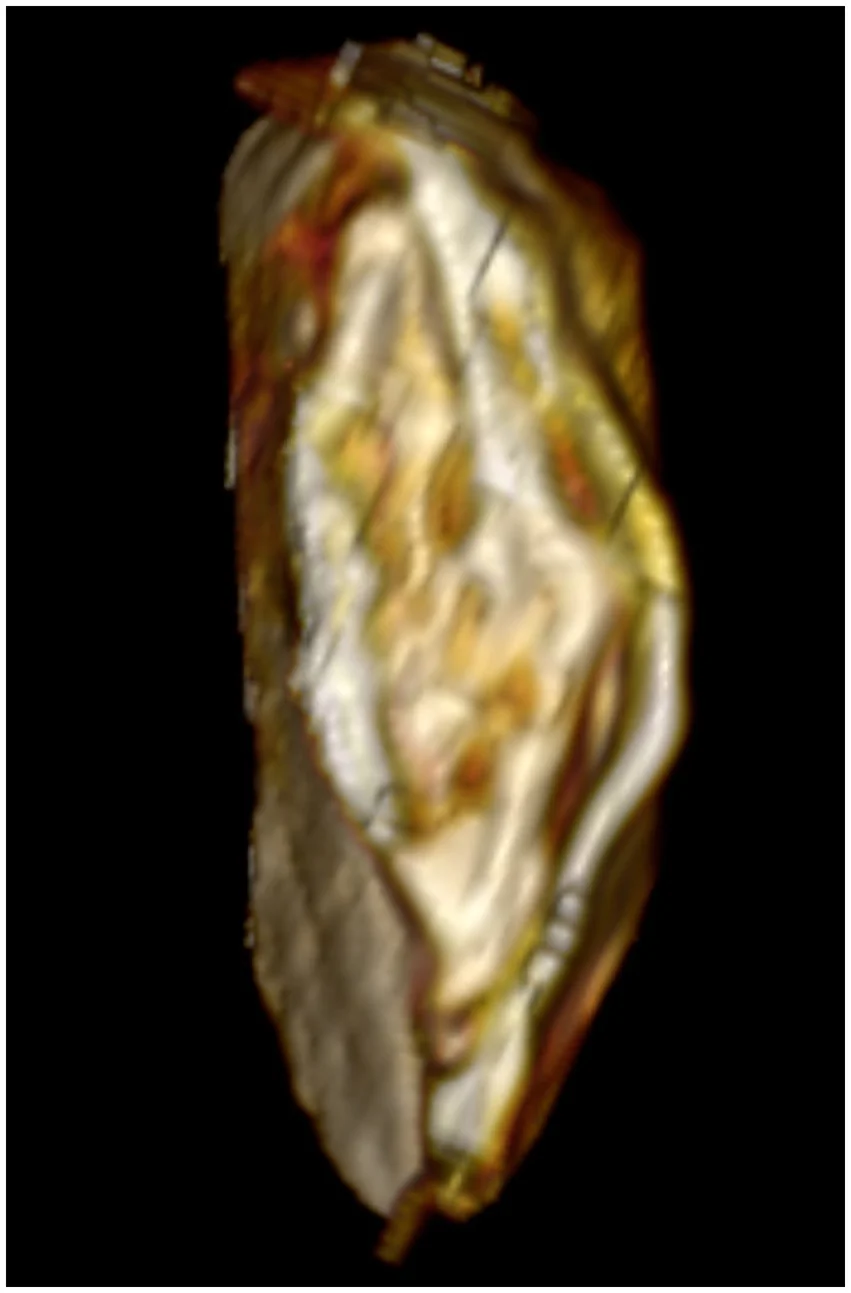
Three-dimensional CT image of the surface of symphysis pubis (left) of anonymous mummified woman referred to as CIT8 indicates stage 5 corresponding to 48.1 years ±14.6. The CIT8 mummy with a screaming face expression was discovered in Luxor, Egypt, near Senmut’s service tomb, the architect during Queen Hatschepsut’s reign (1479–1458 BC).

Computed tomography imaging demonstrated a mature skeleton, confirming full fusion of the epiphyses of all the long bones, as well as the iliac crest apophysis and the medial epiphysis of the clavicle, the latter of which completes fusion in the late twenties. The 3D CT image of the surface of the symphysis pubis is stage 5 according to Suchey-Brooks method corresponding to 48.1 years ±14.6 (14, 15) (Figure 4).

#### 3.2.3 Stature and long bone measurements

The vertex to heel length of the mummy measures 158.2 cm in the sagittal reconstructed CT image. The left and right femur measure 407 and 409 mm, respectively. The left and right tibia measure 34.6 and 34.5 mm, respectively. The left and right humerus measure 291 and 292 mm, respectively. The living stature of the studied woman was estimated from tibial bone length measurements using regression-equations derived from Egyptian population as follows (16):


Staturefromtibiallength=2.699×34.6+61.08=154.4cm±1.921

#### 3.2.4 Pathological changes and cause of death

**Figure 5:**
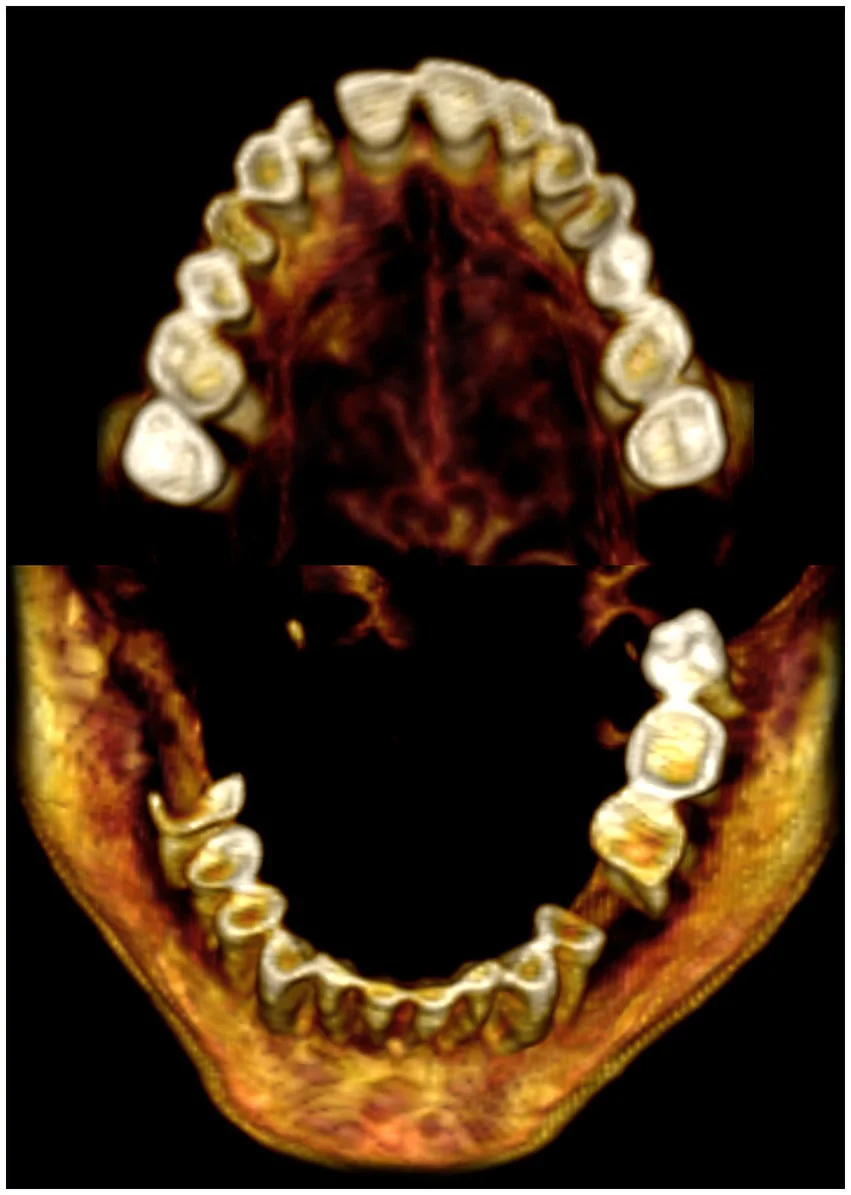
A three-dimensional CT images of the maxillary and mandibular teeth of mummified woman (referred to as CIT8) show that some teeth were missing during life with signs of bone loss (resorption): upper right third molar, upper left third molar; second and third lower right molars, and lower left second premolar. Partially fractured teeth include the right upper lateral incisor and right lower first molar. Mild to moderate attrition of the remaining teeth with no signs of periodontal disease or caries cavities. The mummy CIT8 with a screaming face expression was discovered in Luxor, Egypt, near Senmut’s service tomb, the architect during Queen Hatschepsut’s reign (1479–1458 BC).

- Evidence of mild to moderate teeth attrition. Ante-mortem missing of the following teeth: upper right third molar tooth, upper left third molar; second and third lower right molars and lower left second premolar tooth. There is evidence of bone loss (resorption) of the sockets of the missed teeth. Partially broken teeth: right upper lateral incisor and right lower first molar tooth. There was no evidence of periodontal disease or caries cavities (Figure 5).
- Mild degenerative changes of the joints including the spine. There is mild osteophytic lipping of hip and knee joints. Mild posterior osteophytes at cervical C3–4 disk level; mild anterior osteophytes at cervical C5–6 disk level; Schmorls’ nodules located at lumbar L1–2 disk level; mild anterior osteophytes at L3–4 disk level.
- No CT evidence of atherosclerosis.
- The cause of death was not determined by the CT examination.

#### 3.2.5 Mummification

**Figure 6:**
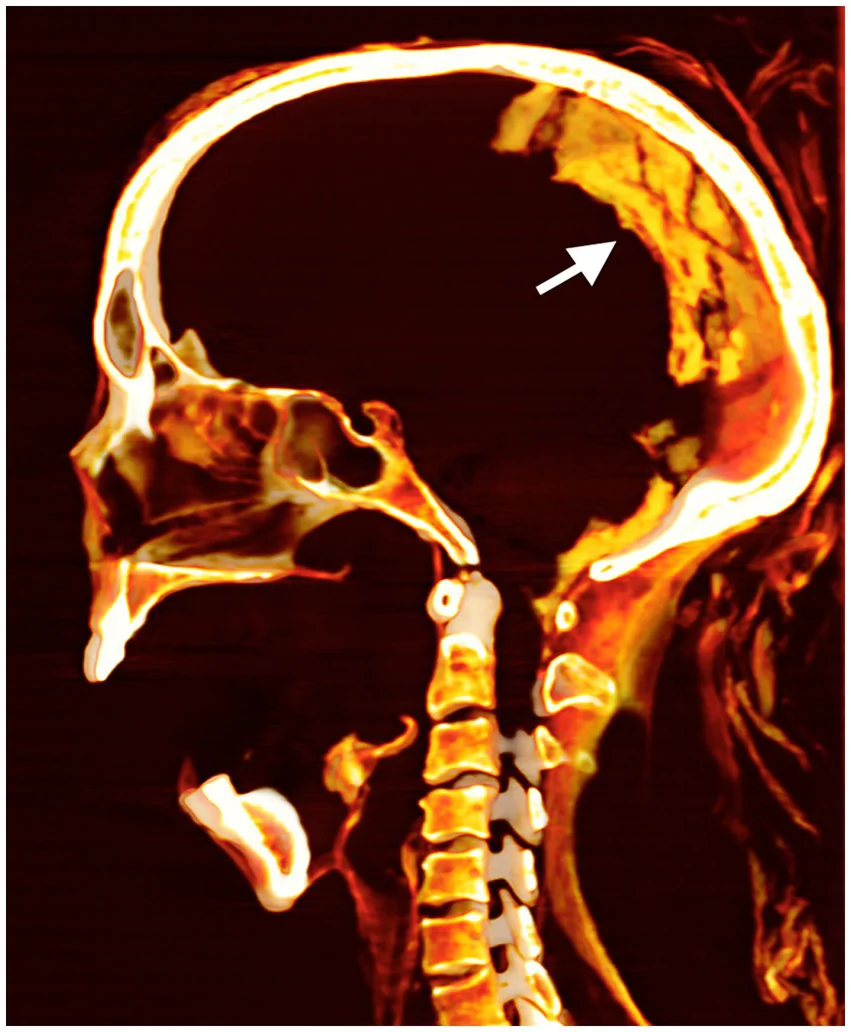
The mid-sagittal three-dimensional CT image of the head of anonymous mummified woman (referred to as CIT8). It shows an unbroken cribriform plate, with the preserved desiccated brain resting at the back of the skull (arrow). The mummy with a widely open mouth was found at Luxor, Egypt, near Senmut’s service tomb, the architect during Queen Hatschepsut’s reign (1479–1458 BC).

**Figure 7:**
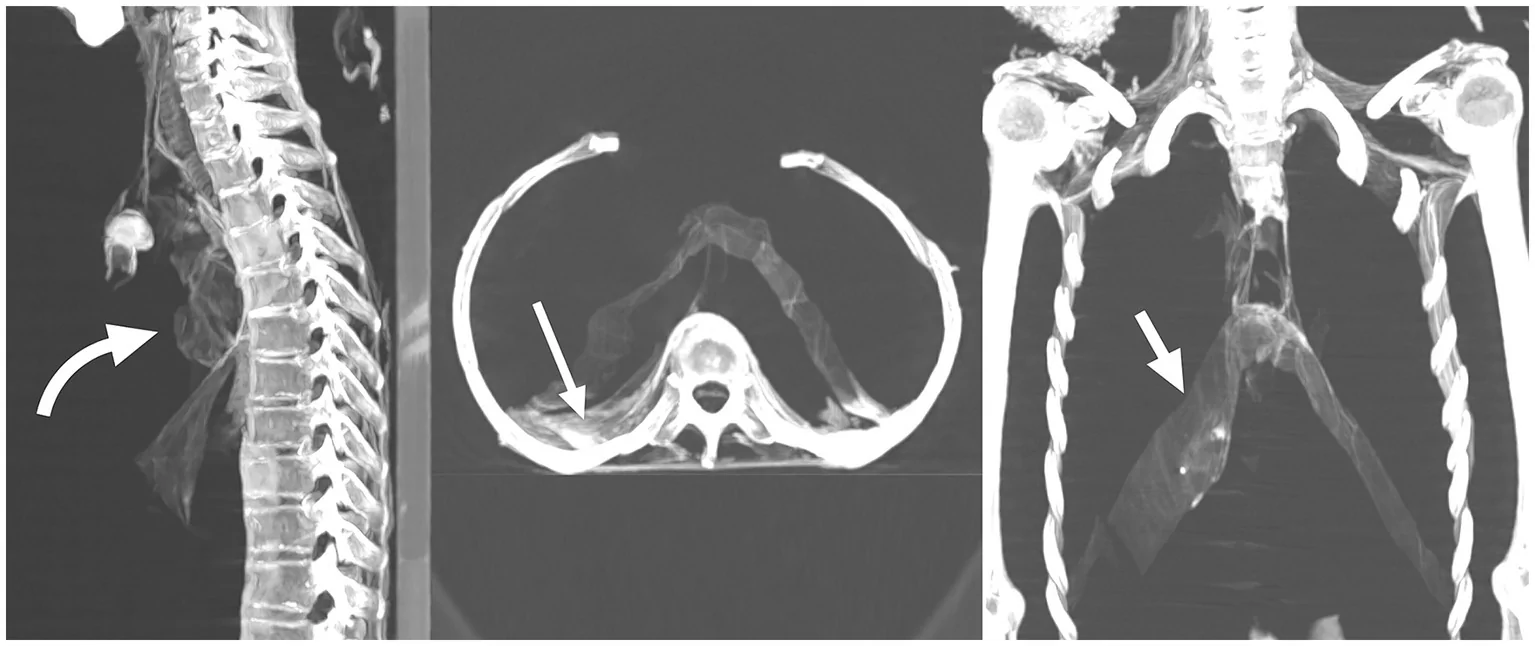
Multiplanar two-dimensional CT images of the torso of mummified woman CIT8 in sagittal, axial and coronal planes show presences of desiccated viscera: heart (curved arrow), abdominal viscera and liver (long straight arrow), and diaphragm (short straight arrows). Findings show no evisceration was done supported by the absence of an evisceration incision. The mummy was found at Luxor, Egypt, near Senmut’s service tomb, the architect during Queen Hatschepsut’s reign (1479–1458 BC).

**Figure 8:**
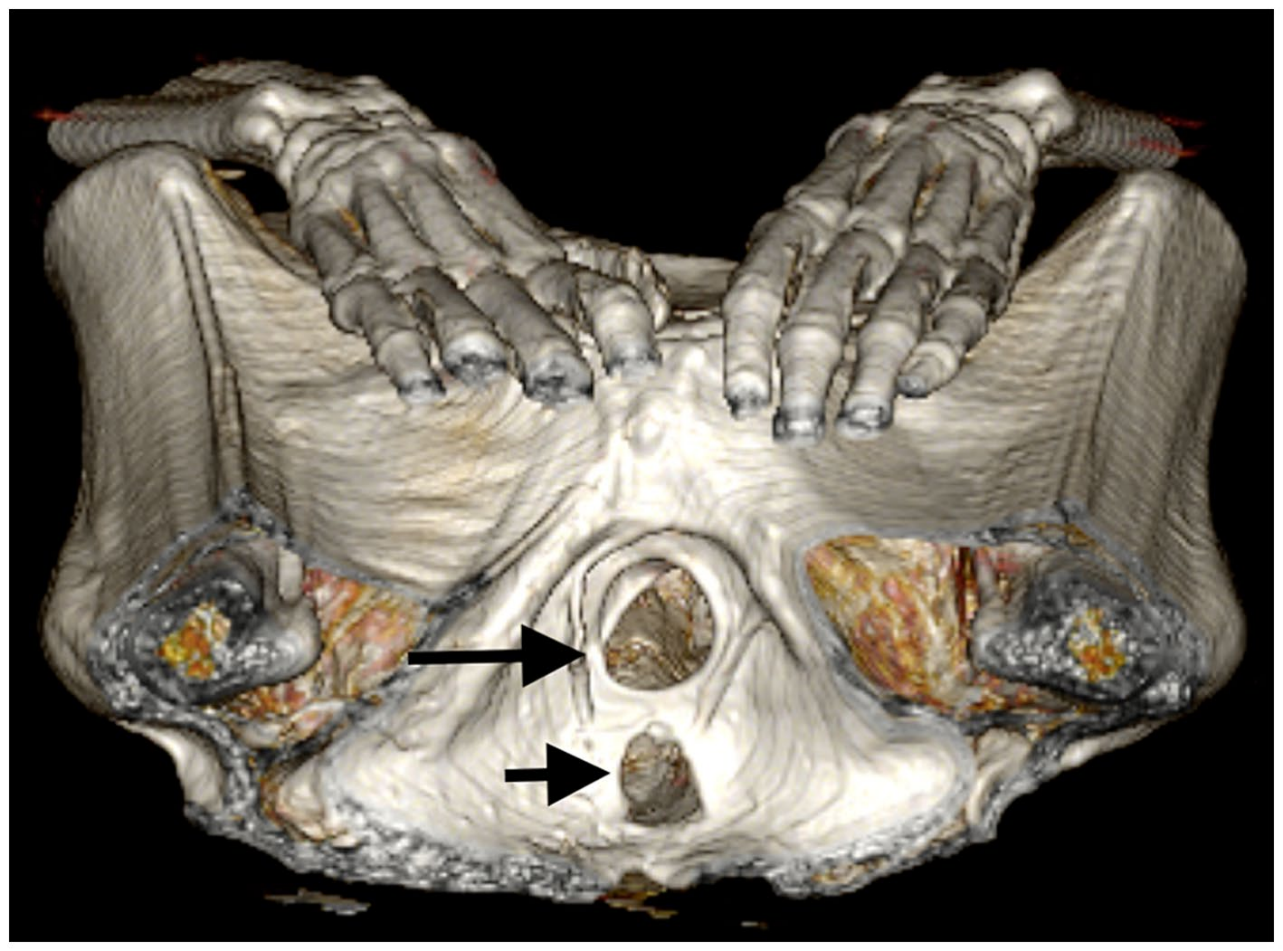
Three-dimensional CT image of the perineal region of anonymous mummified woman (referred to as CIT8). The image shows a considerably dilated vaginal (long arrow) and anal (short arrow) orifices suggests that packing was used to plug the openings (currently non-available). The mummy was found at Luxor, Egypt, near Senmut’s service tomb, the architect during Queen Hatschepsut’s reign (1479–1458 BC).

**Figure 9:**
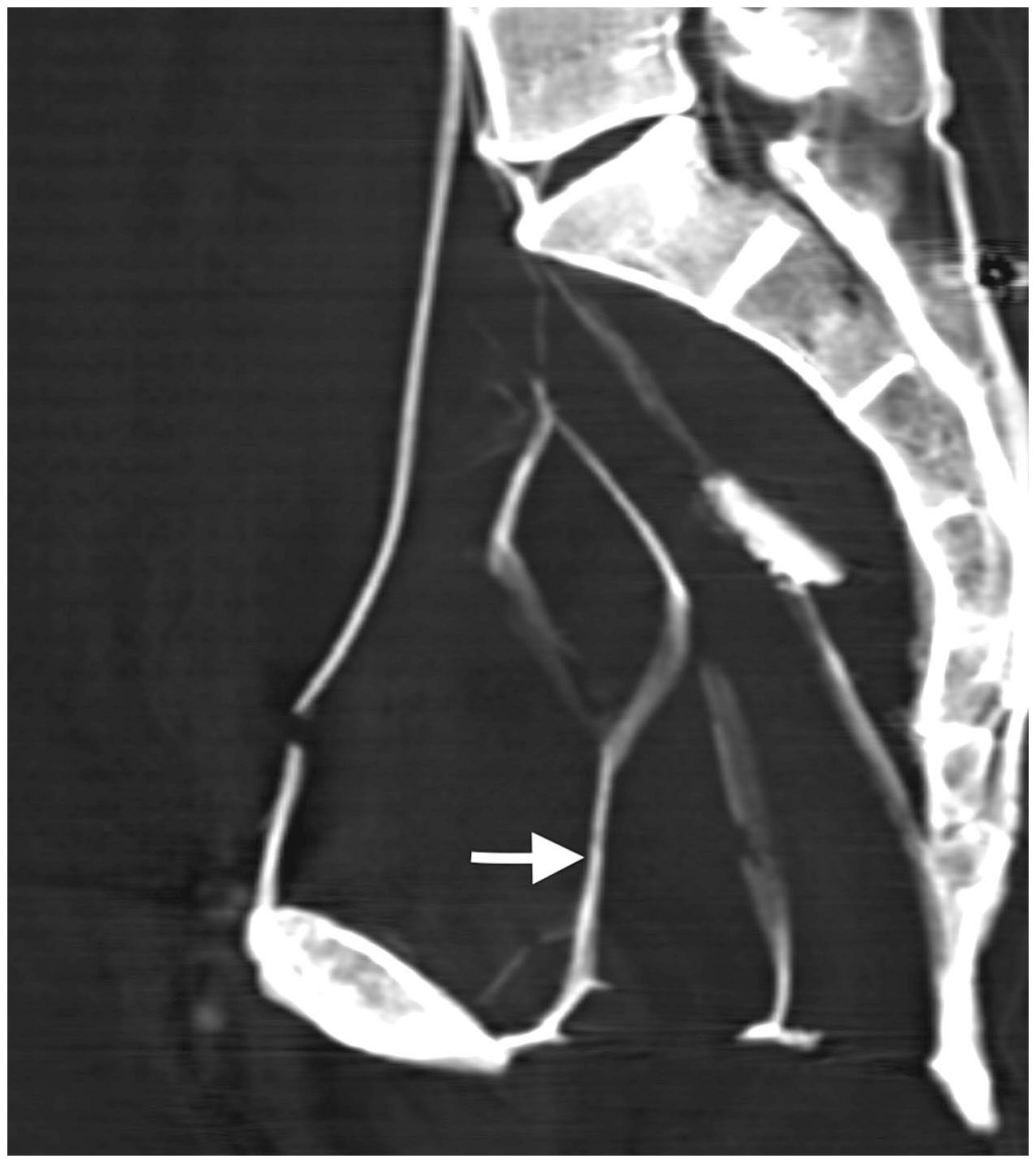
A midsagittal CT image of anonymous mummified woman (referred to as CIT8) shows desiccated strands of pelvic structures (arrow) within the pelvic cavity leading to widened apertures. The mummy was found at Luxor, Egypt, near Senmut’s service tomb, the architect during Queen Hatschepsut’s reign (1479–1458 BC).

- The head is slightly tilted to the left side with a wig on. The mouth is seen widely open. The desiccated tongue is seen at the back of the mouth; no foreign materials or packs could be seen within the mouth.
- Intact skull is noted with no evidence of brain removal (excerebration) attempt. The desiccated brain occupies the posterior part of the skull cavity (Figure 6). There is no evidence of any foreign materials or packing in the orbits, nostrils, or ear openings.
- The interior of the torso contains shrunken desiccated structures including diaphragm, heart, and liver. Streaks of dried tissues are seen in the pelvic cavities. Torso cavity does not contain any foreign materials or packs. There are no openings or embalming incisions could be detected (Figure 7).
- The perineal orifices are enlarged: vagina measures 35 mm × 45 mm in transverse and anteroposterior directions, respectively and anus measures (23 mm ×33 mm in transverse and anteroposterior measurements, respectively). Desiccated strands of pelvic structures are seen within the pelvic cavity (Figures 8, 9).
- No subcutaneous packing, amulets or jewelry could be noted in the mummy.

#### 3.2.6 CT appearance of the wig

**Figure 10:**
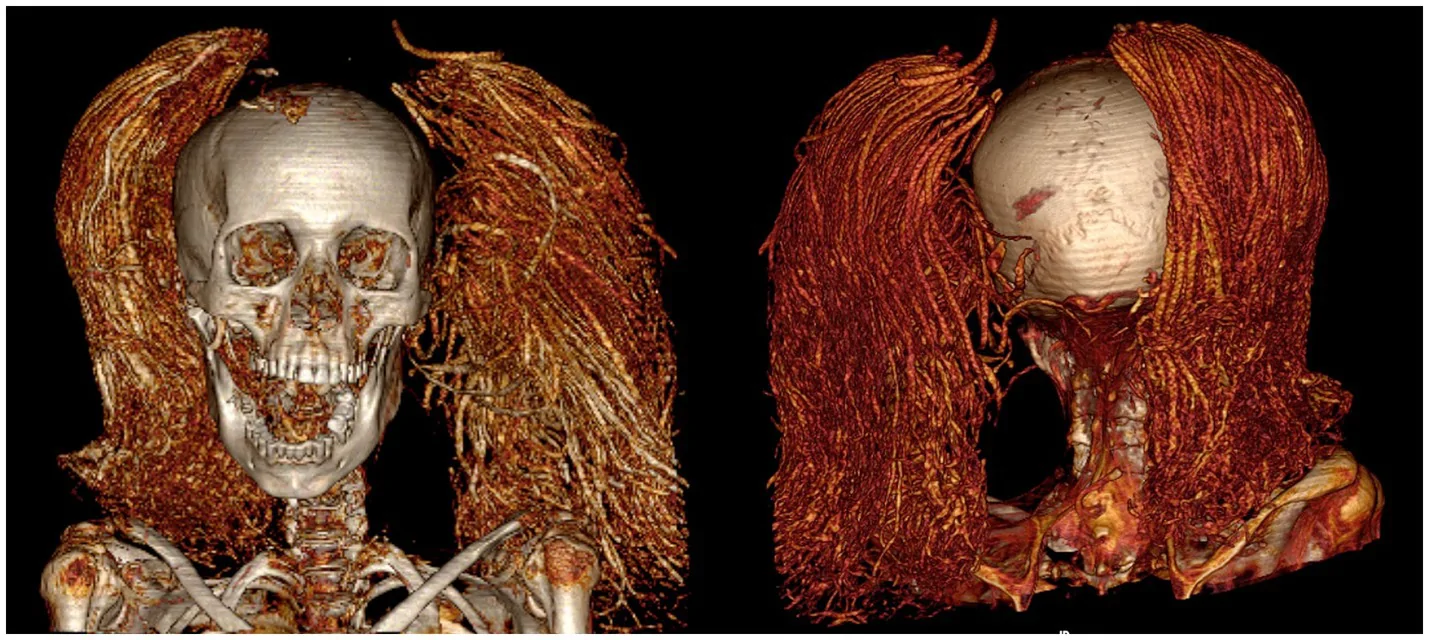
Three-dimensional CT images in front and back views of the wig on the head of anonymous mummified woman referred to as CIT8. The wig is made up of long braids arranged in two widely spaced portions on both sides of the head. CT scanning allowed for image the back of the head without having to physically flip the mummy. The mummy CIT8 was found at Luxor, Egypt, near Senmut’s service tomb, the architect during Queen Hatschepsut’s reign (1479–1458 BC).

**Figure 11:**
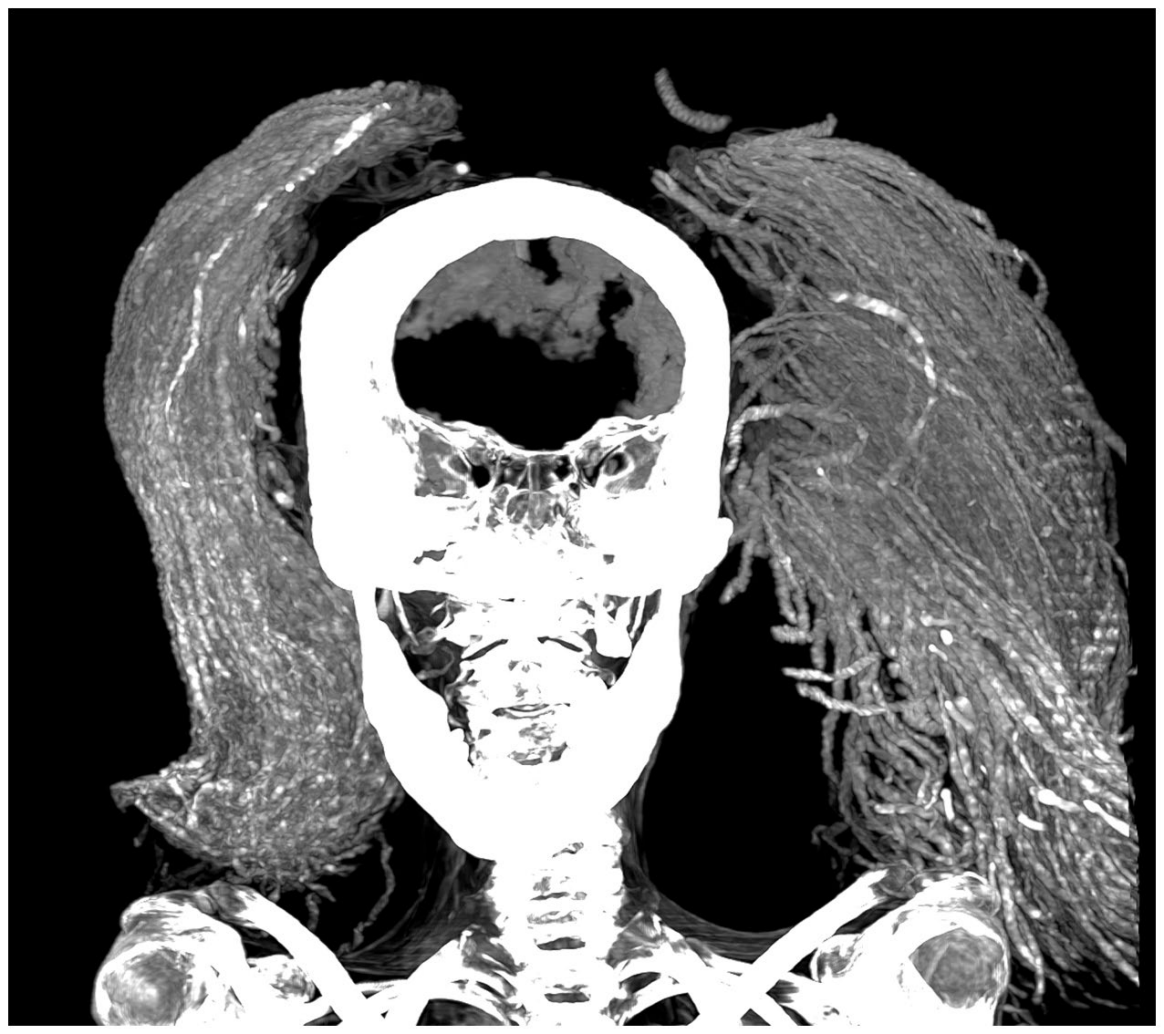
Two-dimensional CT image in coronal plane of the head of anonymous mummified woman referred to as CIT8. The mummy was discovered in Luxor, Egypt, near the service tomb of Senmut, the architect during Queen Hatschepsut’s reign 1479–1458 BC. The wig is comprised of multiple low-density fibers partially coated with dense embalming material, which corresponds to the inspected black consolidated material.

Computed tomography images of the head of the mummified woman CIT8 depict a wig made up of two widely spaced portions on both sides of the head. The wig is formed of several fine braids that extend on both sides to the shoulders level with maximum length of 320 mm (Figure 10). The braids have an average thickness of 2.4 mm and a low density of 15 HU on average. Some braids are covered with dense material that measures 550 HU in average (Figure 11).

### 3.3 Investigations of the mummy samples

#### 3.3.1 Scanning electron microscope

**Figure 12:**
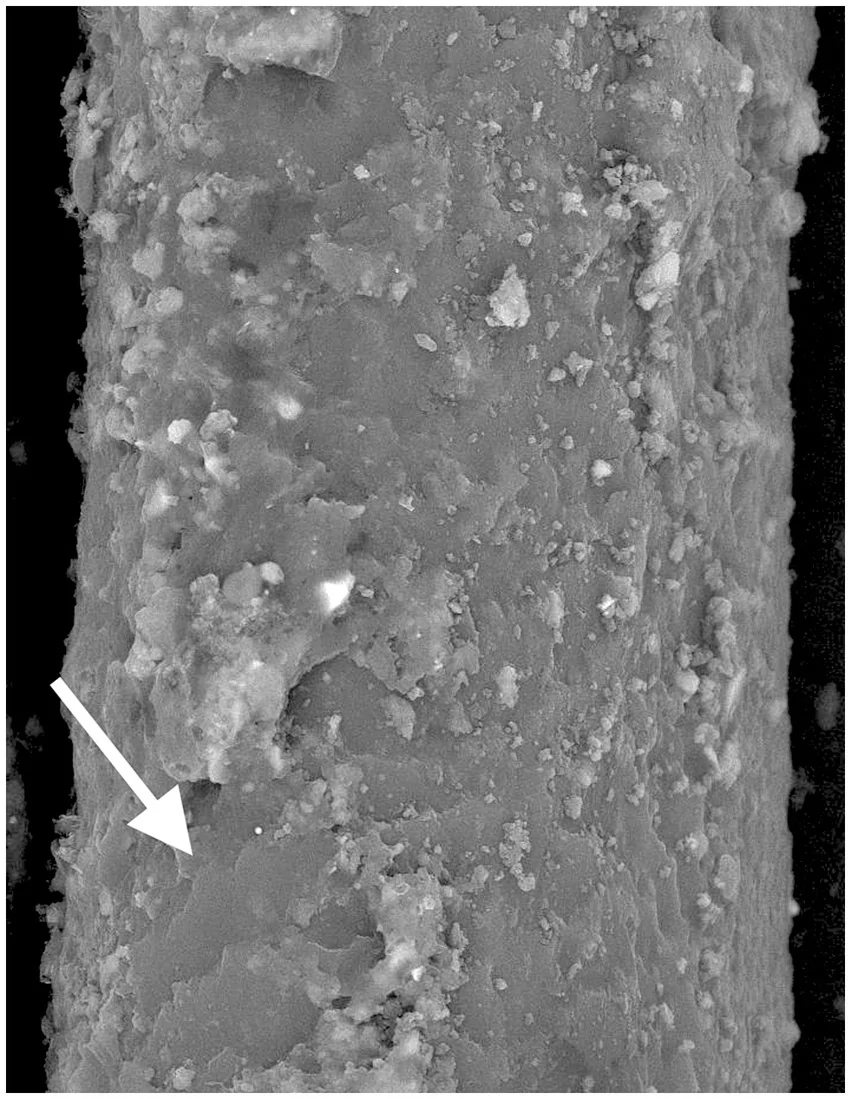
Scanning electron microscope (SEM) magnification 3,000X picture of a hair sample obtained from the front of the head of the mummy CIT8 identifies a human hair with its characteristic layers of flat overlapping structures that resembled scales (arrow) heavily coated with material. This anonymous mummy (known as CIT8) was discovered in Luxor, Egypt, near Senmut’s service tomb, the architect during Queen Hatschepsut’s reign 1479–1458 BC.

**Figure 13:**
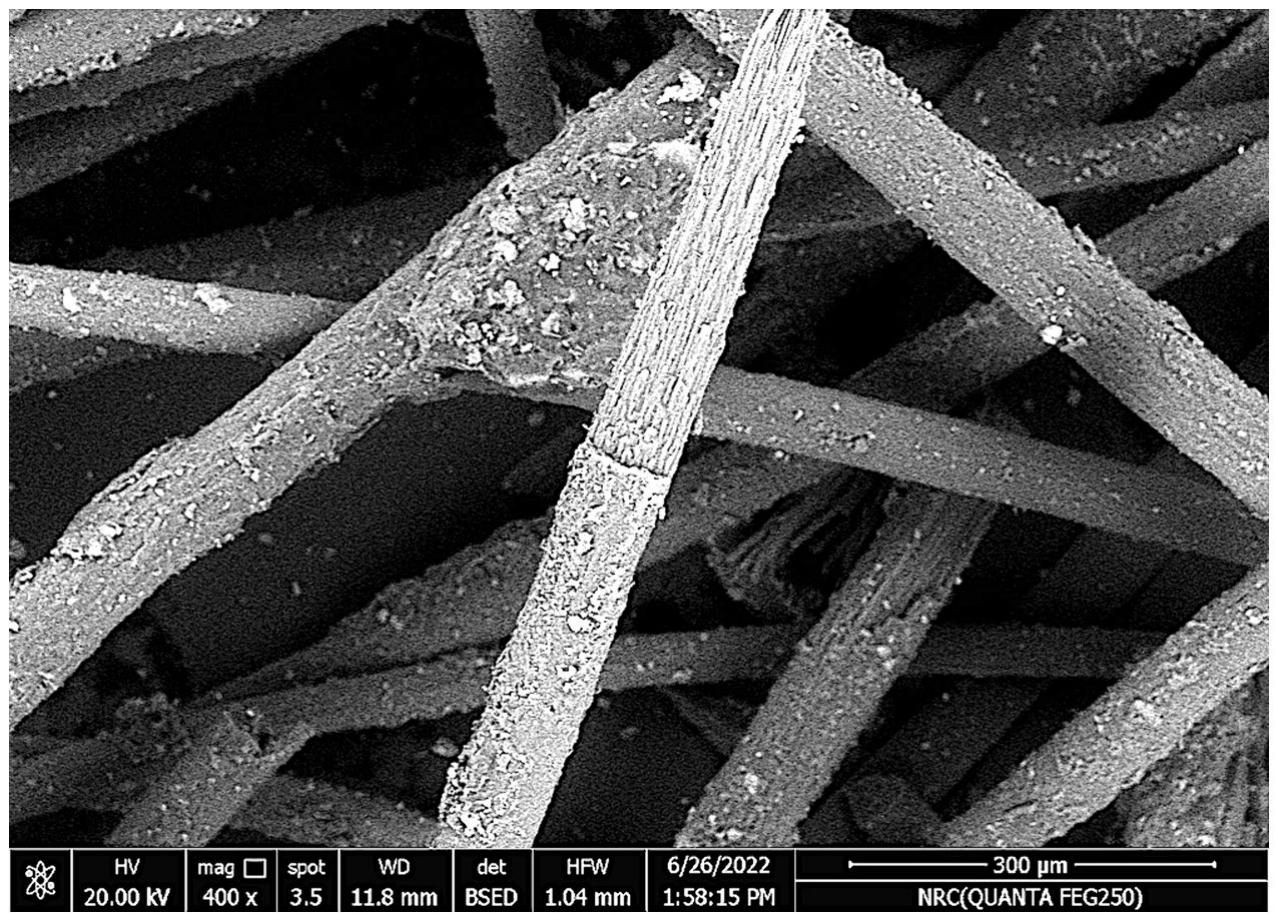
Scanning electron microscope (SEM) magnification 400X picture of a wig fibers sample on mummy’s CIT8 head. The fibers of the wig appear cylinders devoid of animal or human hair features suggesting plant fibers. The anonymous mummy (known as CIT8) was discovered in Luxor, Egypt, near Senmut’s service tomb, the architect during Queen Hatschepsut’s reign 1479–1458 BC.

We examined the natural hair and wig samples using SEM. The hair sampled from the front of the mummy’s head was determined to be human with its characteristic layers of flat, overlapping structures that resembled scales (18) (Figure 12). The fibers of the wig showed cylinders devoid of animal or human hair features suggesting plant fibers (Figure 13).

#### 3.3.2 Fourier-transform-infrared-spectroscopy

Analysis using FTIR for the skin sample identified embalming materials with absorption curves corresponding to juniper and frankincense.

Fourier-transform-infrared-spectroscopy analysis of the natural hair surface revealed that it is heavily coated with henna and juniper.

**Figure 14:**
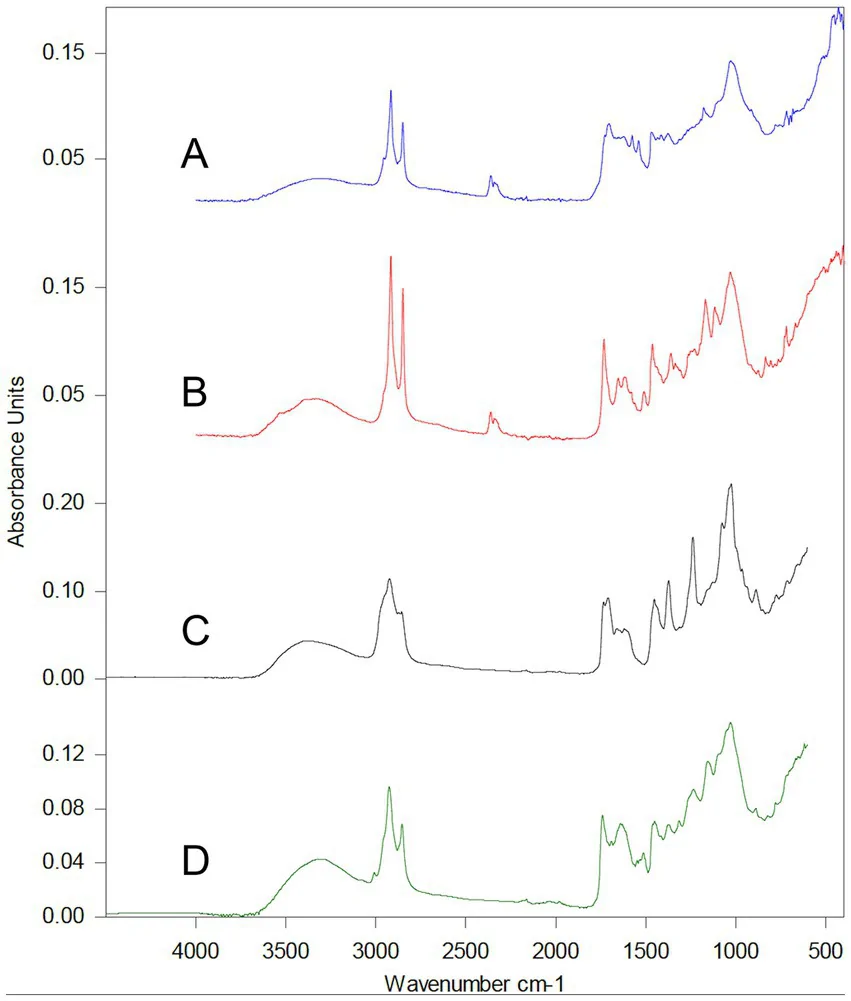
Fourier Transform Infrared spectroscopy (FTIR) of a sample from the wig fibers on the head of the mummified woman CIT8 shows the absorption curves identify the material used to manufacture the wig fibers and the embalming ingredients on the surface of the wig fibers. Absorption curve **(A)** is for the wig hair fiber sample, which matches with curve **(B)** for the palm fiber (midrib) standard. Absorption curves **(C,D)** correspond to frankincense and juniper embalming ingredients, respectively, detected on the surface of the sampled wig fibers. The anonymous mummy (known as CIT8) was discovered in Luxor, Egypt, near Senmut’s service tomb, the architect during Queen Hatschepsut’s reign 1479–1458 BC.

Fourier-transform-infrared-spectroscopy identified the wig hair through its characteristic absorption curve as fibers of midrib of date palm based on the characteristics of lignocellulosic fibers peak observed at 3200–3600 (19). FTIR curves identified frankincense and juniper material on the surface of the wig fibers sample (Figure 14).

#### 3.3.3 X-ray-diffraction-analysis

X-ray-diffraction-analysis examination of the sample of wig fibers’ surface identified the black consolidated material as crystalline made up of quartz, magnetite, and high albite. Albite is a form of feldspar mineral that is composed of sodium, aluminum, silicon, and oxygen.

## 4 Discussion

In this work, we examined the mummified woman CIT8 several decades after it was found and placed in storage first at Kasr Al Ainy Faculty of Medicine then at Cairo Egyptian Museum.

### 4.1 Physical examination

At the time of this study, the mummified woman CIT8 has a rectangular defect with sharp, regular margins at the center of the anterior chest wall, as well as collapsed anterior abdominal wall. According to the mummy’s description at the time of discovery in the Metropolitan Museum’s 1935–1936 burial cards, the woman’s torso had undamaged skin.

In 1998, the mummy was photographed after being moved from Kasr Al Ainy Faculty of Medicine to Cairo Egyptian Museum. The photos revealed a defect in the center of the anterior chest wall; however, the anterior abdomen wall had not collapsed. The implication is that the present abdominal wall collapse happened at the Cairo Egyptian Museum possibly from improper storage conditions. We assume that the chest wall defect was inflicted at Kasr Al Ainy Faculty of Medicine during the mummy autopsy. The lack of bone fragments within the chest cavity and the sharp edges of the chest defect, which were probably caused by a sharp cutting tools, lend support to this theory.

Many of excavated mummies in Egypt in the 19th and early 20th centuries were sent to Kasr Al Ainy Faculty of Medicine in Cairo to be examined by the school’s professors, e.g., MA Ruffer (1859–1917), GE Smith (1871–1937), and Douglas Derry (1874–1961) (20). Examination of the mummies sent to Kasr Al Ainy Faculty of Medicine lead to important discoveries in understanding the natural history of diseases such as the discovery of calcified schistosoma egg, identification of tuberculosis of the spine, and arterial atherosclerosis (21, 22). The museum of the Anatomy Department at Kasr Al Ainy Faculty of Medicine in fact still hosts mummies and ancient Egyptian human remains (20).

### 4.2 CT scanning

Computed tomography is the gold standard non-invasive diagnostic imaging method for mummy analysis, providing access to large data sets for detailed morphological examination of mummy’s inner and outer structures. CT scanning allows also for metric measurements and tissue density measurement using Hounsfield Units (HU) (6, 7).

The age of death of the mummified woman CIT8 was estimated to be 48.1 years ±14.6 based on the morphology of the pubis symphysis surface as shown non-invasively by CT images. CT pictures show mild degenerative changes in bones, joints, and spine, which are consistent with the estimated patient’s age of a middle-aged adult.

Computed tomography aids in the precise measuring of the mummy’s height and the length of its long bones (6). The mummy is in a recumbent extended posture. The height of the mummy measured in the sagittal reconstructed CT image from the top of the head (vertex) to heel is 158.2 cm. The estimated living stature of the mummy from long bone lengths using Egyptian-based stature regression formulae (16) was 154.4 cm.

A body’s recumbent length is longer than the individual’s vertical standing measurement. The arrangement of the spine differs between the two positions. The lumbar lordosis relaxes in the recumbent posture, extending the spine, while the intervertebral disks compress when standing upright. Therefore, cadaveric length is not a reliable indicator of living stature. Studies report differences of between 2.35 and 3.68 cm (23–25).

Computed tomography scans have made it possible to identify ancient diseases and get a deeper comprehension of their natural history, including cardiovascular, bone, and dental health conditions (26–28).

The widely opened mouth of mummy CIT8 enabled adequate physical inspection of tooth attrition, as well as broken and missing teeth. Reconstructed CT images of maxillary and mandibular teeth of mummy CIT8 showed with clarity broken teeth, missing teeth associated with bone loss (resorption), and attrition of the remaining teeth, which had been observed in physical inspection.

In previous studies, CT scanning proved beneficial in identifying the state of the teeth in ancient Egyptian mummies. Missing teeth, supernumerary teeth, attrition, peridontal disorders, and dental absecesses were detected using CT scans of mummies (6, 7, 11). However, there was no CT evidence of periodontal disease or caries cavities in mummy CIT8. The multiple missing teeth were most likely lost during life (ante-mortem) since there is evidence of bone resorption, which occurs when a tooth comes out and the socket is left to heal. It is unknown if the missing teeth in the studied mummy were intentionally removed or not. According to hieroglyphic records, the ancient Egyptians practiced dentistry. In fact, the world’s known first record of a dentist was Hesyre (about 2660 BC), a chief dentist in ancient Egypt (29). Some ancient human remains and mummy research have shown instances when teeth extraction may have happened in ancient Egypt (29–31); but other studies threw doubt on it (32). Further research and information on ancient Egyptian dentistry is required. According to preliminary research studies, ancient Egyptians appear to have more dental wear and decay than modern populations (33).

Preservation of the body served a sacred purpose in ancient Egypt, ensuring that the spirit would live forever and enabling it to recognize the body after death (34). The ancient Egyptians noted that the climate of hot, dry deserts inhibited the decomposition of buried corpses. The soft tissues of the dead body dried out and desiccated in the hot Egyptian desert, halting the bacterial growth that would have otherwise degraded the body. In ancient Egypt, artificial mummification using inducing techniques and embalming ingredients led to increasingly complex body preservation over the course of 30 centuries (29).

Very sparse ancient literature now remains about the techniques of mummification mainly from accounts of early travelers, like the Greek traveler Herodotus (484–425 BC) (6, 34). Herodotus stated that the mummification process varied depending on the financial status of the deceased. Wealthy citizens had the most lavish treatment, while for the middle and poorer classes; mummification was downgraded or minimally performed. Mummification practices thus reflect social and political order (17, 35).

The classic method of mummification in the New Kingdom of ancient Egypt (1550–1069 BC) included the following steps: excerebration (removal of the brain) through a defect created in the skull base by an iron hook inserted through the nostrils; evisceration (removal of the internal organs) from a lower abdominal incision, with the exception of the heart; cleaning the body cavities, dehydrating the body with natron salt, packing the body cavities with embalming agents, placing amulets, and covering the body with embalming materials (34, 35).

The body of mummified woman CIT8 was positioned in a recumbent extended posture with her hands on her groin consistent of non-royal women burials in New Kingdom. This differs from royal women’s arm positions, in which the left arm is flexed across the chest and the right at the side (36).

By revealing the bone and the residual tissues, as well as the materials and packs inside the mummified body, CT imaging can assist in determining the mummification style (6).

Computed tomography scan of the mummified woman CIT8 shows no symptoms of evisceration; the torso cavity retains the desiccated viscera scoring 70% with Aufderheide’s visceral index, suggesting good preservation.

The early mummy excavation records, which describe the body’s undamaged skin, mention that there was no evisceration incision (4). The mummified body in this report exhibits dilated anal and vaginal perineal orifices (34). However, in other natural desiccations, the perineal openings are often collapsed (17, 34). The dilated, rounded perineal apertures indicate that some kind of packing was likely utilized to plug the openings or to capture and contain any bodily fluids exiting these locations. Pelvic CT images of the mummified Thoya, mother-in-law of King Amenhotep III (1390–1352 BC), from the 18th Dynasty, indicate artifacts within the dilated orifices of the vagina and rectum. The emablmers were supposed to have placed these artifacts as a stopper to close the orifices and prevent leaks (6). However, because there are no artifacts in the pelvic orifices of mummy CIT8, we hypothesize that the “plugs” were removed long after desiccation, resulting in larger and dry openings.

The embalmers did not excerebrate the mummy since CT scans showed the skull base’s integrity and the presence of a dry shrunken brain at the back of the cranial cavity CIT8. Although excerebration is seen in those who benefited from the New Kingdom’s classic mummification, CT scanning revealed that brain removal was not performed on royals from the early 18th Dynasty (1550–1295 BC), including Amenhotep I (1525–1504 BC), Thutmose II (1492–1479 BC), and Thutmose III (1479–1425 BC) (37, 38).

Ancient Egyptians wore amulets and jewelry for protection during life and after death, often resembling gods, animals, or other symbolic objects (8). Embalmers used these objects to beautify corpses, placing them on the mummy or in between the body wrappings (39). CT offers a detailed analysis of these objects, determining their location, form, and size (40). The CT images of a mummified woman in this study showed no amulets or jewelry, but excavation notes mentioned she was wearing two rings with jasper scarabs set on gold and silver rings which are displayed currently at the Metropolitan Museum in New York (4) (Supplementary Figure 1). The scarab beetle was a symbol of resurrection in ancient Egypt (39). The material used for these amulets and jewelry varied depending on the person’s wealth, with expensive items often indicating socio-economic status.

Wigs (fake hair) and hair extensions worn by live people, as well as burial apparel, illustrate ancient Egyptians’ desire for ornate and beauty (41). Wigs were often used in ancient Egyptian funerary rites. The oldest wig was discovered in Naqada II’s (about 3600 BC) burial site at Hierakonpolis (38), while another was discovered in Hefefi’s tomb at El-Hagarsa (c. 2160–2055 BC) (39). The latter wig was fashioned of long, straight braids of twisted flax fibers sealed with resins and beeswax.

The mummy in this study had a wig on its head that is made up of two widely apart sections. The wig’s two parts were seen connected on the mummy’s head in the 1939 photos (Figure 1); this indicates current deterioration of the conservation state of the wig since that time.

Some mummified women in ancient Egypt were buried wearing wigs. Princess Hentempet (c. 1500 BC) and Meryt (1400 BC) were buried with wigs of different styles. Hat-Nufer, mother of Senmut (from Hatschepsut reign 1479–1458 BC), whose mummified body was discovered near tomb TT71, adjacent to the burial site of mummified woman CIT8, her mummy’s short gray curls had been extended by connecting tapered plaits of dark brown hair (41).

Computed tomography is both a qualitative and quantitative imaging method. In addition to identifying the morphology of the mummy, CT enables density measurement using Hounsfield Units (HU), which vary depending on X-ray diffraction in different tissues and structures. The results are compared to existing database of structures and materials (6). The CT scan of the wig in this investigation reveals that it is composed of multiple low density (15 HU) spiral braids that were partially covered by dense embalming material measuring (550 HU), which corresponded to the inspected black solidified material.

### 4.3 Scientific investigations of mummy samples

Further scientific analysis with FTIR and SEM revealed that the wig was composed of plant fibers from the date palm midrib. In ancient Egypt, wigs were constructed from human hair, linen, or date palm fiber (42). The specifics of the wig-making process in ancient Egypt were unknown, until a wigmaker’s workshop dating to between 1800 and 1700 BC was found at Deir el-Bahari. The workshop contained wigs in different stages of production (43, 44).

In this study, XRD examination identified the CT dense black consolidating material on the surface of the sampled wig fibers as crystalline made up of quartz, magnetite, and high albite. This material is similar in composition to fine dark powder of manganese dioxide and quartz found in a small sachet in the wigmakers workshop at Deir el-Bahari and was sprinkled over the hair for cosmetic reasons (44). Combining CT pictures with scientific testing (SEM, FTIR, and XRD) resulted in more detailed understanding of ancient Egyptian wig structure and materials used.

The mummification process is covered in very few documented ancient Egyptian sources, such as the Ritual of Embalming papyri, and none of these writings specifies the precise ingredients that are required to prepare the embalming materials (45). The chemical composition of several embalming agents used in ancient Egypt has recently been identified by scientific analytical studies (46, 47).

Fourier-transform-infrared-spectroscopy analysis revealed that skin, hair, and wig samples were treated with embalming materials, with juniper resin being the primary component. Additionally, frankincense was found in the skin and wig samples, whereas henna was found in the natural hair sample.

Resin is anaturally occurring plant substance, mostly made by some trees (47). The resin known as juniper comes from the *Juniperus phoenicea* tree, which grows in Syria and the eastern Mediterranean (48). Frankincense is an aromatic gum-resin substance obtained from *Boswellia papyrifera* tree comes from three distinct regions: East Africa (Somalia and Sudan), Southern part of the Arabian peninsula (Yemen and Oman), and North-Western India (49). Ancient Egyptian sources indicate that Queen Hatshepsut brought frankincense from land of Punt (likely Somalia in Africa). Frankincense and juniper were found in the tomb of Tutankhamun (17, 48).

After being heated, resin becomes a liquid that solidifies upon cooling. Resins were utilized by Egyptian embalmers because they are not water soluble and have an anti-insect-pathogens properties that assist seal the mummy’ surface and prevent degradation (47, 48). The analysis of the remains that were found in a vessel labeled “antiu” that was found in a mummification workshop in Saqqara showed that it was made of juniper resin mixed with plant oils and animal fats (47).

Ancient Egyptians cultivated henna plant (*Lawsonia alba* and *L. inermis*). Ancient Egyptians colored their mummies’ and live people’s hair with henna reddish-brown leaf extract, as a cosmetic. Ointments and perfumes have also been made with henna as a component (17).

In this study, juniper was found in both natural hair and wig’s fiber samples. It is known that ancient Egyptians applied juniper ointment on their hair. Juniper’s natural dark color was probably employed as a dye to partially penetrate and cover the hair. Black hair color was perhaps the most popular choice for ancient Egyptians to regain their youthful look (42).

Desiccation is the process used in ancient Egyptian mummies to preserve soft tissue. This process can be carried out in a few different ways, such as natural mummification by burial in a hot desert or by using natron salt and treating the body’s surface with embalming materials like resin (17).

The use of CT imaging technology in the current study yielded important information regarding the mummy’s funerary treatment. Lack of evisceration in mummy CIT8, a procedure often associated with traditional New Kingdom mummification, implied that the embalming procedure was of low quality and that soft tissue preservation was achieved through natural mummification.

Nevertheless, imaging techniques like CT scans cannot identify the embalming substances that were utilized during mummification (46). Advanced analytical tests of the mummy’s samples revealed the use of embalming materials, such as juniper resin, shedding light on the higher quality of the mummification process and dismissing the earlier theory of poor natural mummification based only on CT scan results (46). The dead body in ancient Egypt could be preserved from the harmful destructive effect of bacteria and insects by using embalming materials like juniper and Tea Tree Oil, which possessed antibacterial and insecticidal properties (17, 48–51). This style of mummification that lacked evisceration but used embalming materials on the surface of the mummy was reported in two mummified bodies adorned with gold dated to 18th dynasty found in tomb in Deir el Medina, Luxor belonged to wealthy architect Kha and wife Merit (46). The use of costly, imported embalming ingredients, such as frankincense and juniper, may indicate the quality of mummification and provide insight into the historical embalming material trade (47).

### 4.4 Open mouth appearance and cause of death

With her mouth open wide, the mummified woman in this study CIT8 looks to be screaming. The mechanism of opening the mouth is controlled by the temporo-mandibular joint, which is maintained by muscles and ligaments that connect the mandible to the skull. Opening of the mouth occurs when these muscles relax during sleeping or when they decompose after death (52). In order to keep the deceased’s mouth closed, embalmers frequently wrapped the mandible around the skull (53).

Occasionally ancient Egyptian mummies were discovered with their mouths open (6). Two royal mummies from the Deir el-Bahari cache, a man and a woman, have their mouths widely opened as if they are screaming. The man was identified as Pentawere, a 20th Dynasty prince who was put to death for setting up the Harem Conspiracy, a plot to assassinate his father Ramesses III (1185–1153 BC) (6). Pentawere’s body was barely embalmed, which may indicate that the embalmers neglected to keep his mouth closed (8).

The other mummy (also known as Unknown Woman A) had inscriptions on the wrappings read, “The Royal daughter, royal sister Meritamun,” most likely referring to the daughter of King Seqenenre Taa II and sister of King Ahmose (1533–1525 BC) from the beginning of the 18th Dynasty. In contrast to the typical mummy pose of a stretched-out body with straight legs, mummified Princess Meritamun’s head was tilted to the side and her legs were crossed. The CT scan of the mummified Princess revealed significant coronary artery atherosclerosis, suggesting that she likely suffered a major myocardial infarction that caused her sudden death (9). Although cardiovascular disease is likely a contributing factor to the individual’s health, it may not be the cause of death. Meritamun’s mummy widely opened mouth was probably caused by a postmortem normal jaw drop that was likely maintained by rigor mortis (54).

Rigor mortis is the stiffening of muscles and joints after death, which begins with the face and lasts for several hours. Rigor spreads throughout the muscular mass, peaking between 6 and 12 h. This state then remains constant until the muscle mass begins to autolyze. Full rigor can last for 18–36 h before fading (55). Ancient Egyptian mummification procedures helped preserve the deceased body from decay (34). Embalmers likely mummified the contracted body of the Princess before it decomposed or relaxed, thus preserving her posture at death. It is possible that the contracted muscles must have prevented embalmers from closing the mouth (9).

Unlike the mummified bodies of Pentawere and Meritamun, the CT scan of the screaming mummy CIT8 did not show cause of death or explain her physical appearance (8, 9). The funerary techniques the embalmers employed on the corpse of mummy CIT8, including the use of a wig, rings, pricey imported embalming materials, and placing the mummy in a wooden coffin, indicating good mummification quality. This makes it less plausible that the embalmers were careless to secure the mouth closed.

Cadaveric spasm is another type of spasm that demonstrates the final action prior to death.

It occurs after severe physical or emotional activity, leading to immediate postmortem rigor as the contracted muscles become rigid immediately following death and are unable to relax. The phenomenon is rare observed mainly in forensic pathology and not fully understood; it could be caused by motor nerve activation, but for some reason, normal relaxation fails. Unlike rigor mortis, cadaveric spasm affects only one group of muscles, not the entire body (55, 56).

Based on its burial location beneath TT71 service tomb of Senmut, this anonymous woman (referred to as CIT8) is thought to be related to Senmut (3, 57). The mummy’s screaming facial expression in this study could be read as a cadaveric spasm, implying that the woman died screaming from agony or pain.

The scientific debate includes that postmortem muscle spasm dissipating after 18–36 h. Similar to the condition of the mummified princess Meritamun, embalmers likely mummified the contracted body of the woman known as CIT8 before it decomposed or relaxed, thus preserving her opened mouth position at death. It is also possible that the contracted muscles must have prevented embalmers from closing the mouth (9).

Yet, according to some academics, a mummy’s facial expression does not necessarily indicate how they were feeling when they died. Aufderheide asserts that the putrefaction process, the rate of desiccation, and the compressive force of the wrappings could all affect a mummy’s facial expression. Burial procedures or post-mortem alterations might have contributed to the phenomena of mummies with screaming appearances (17).

The true history or circumstances of the death of the woman known as CIT8 are unknown; hence, the cause of her screaming facial appearance cannot be established with certainty. Similarly, the fate of Senmut remains unknown; he abruptly left public life between Hatchepsut’s regnal years 16 and 20 and was never interred in either of his elaborately planned tombs (TT71 or TT353) (58).

## 5 Conclusion

Combining the advantages of paleoradiology with scientific analytical investigations provided enhanced comprehension of the screaming woman mummy from Senmut’s relatives’ burial beneath Theban tomb TT71. CT scanning non-invasively revealed the mummy’s inner and outer morphology, enabled metric and tissue densities measurements, and estimation of age at death as 48 years. It also evaluated the mummification technique based on retained viscera and absence of embalming packs. Advanced analytical studies revealed that embalmers utilized expensive imported materials such as Juniper resin, contradicting the traditional belief that the non-removal of the viscera implied poor mummification, resulting in careless embalmers sealing the mouth. Combining CT images with the scientific testing resulted in more detailed understanding of ancient Egyptian wig structure and material composed of date palm midribs coated with crystalline material and frankincense.

The widely opened mouth could be a result of a facial expression of suffering before death, fixed by cadaveric spasm. The study also explores how rigor mortis, cadaveric spasms, tissue decomposition, burial techniques, and postmortem alterations may contribute to a mummy’s screaming appearance.

## Data availability statement

The datasets presented in this article are not readily available because the data collected during the current study are available from the corresponding author on reasonable request and with permission of the Egyptian Ministry of Tourism and Antiquities. Requests to access the datasets should be directed to saharsaleem@cu.edu.eg.

## Ethics statement

The study was approved by the Egyptian Ministry of Antiquities and Tourism. Ethics reference number: Not applicable. Authors confirm adherence to ethical standards concerning the handling of human remains.

## Author contributions

SS: Conceptualization, Data curation, Formal analysis, Funding acquisition, Investigation, Methodology, Project administration, Resources, Software, Supervision, Validation, Visualization, Writing – original draft, Writing – review & editing. SE: Conceptualization, Data curation, Formal analysis, Funding acquisition, Investigation, Methodology, Validation, Visualization, Writing – review & editing.

## Footnotes

^1^ https://www.metmuseum.org/art/collection/search/549021
