# Supplementary material for: Paleoradiological and scientific investigations of the screaming woman mummy from the area beneath Senmut’s (1479–1458 BC) Theban tomb (TT71)
Source: Front Med (Lausanne). 2024 Aug 2;11:1406225. doi: 10.3389/fmed.2024.1406225 (PMC11328696; doi:10.3389/fmed.2024.1406225)
Supplement: Supplementary file 1 [file Data_Sheet_1.docx]

Supplementary Material

**Supplementary Figures**

**Legends**

**Supplementary Figure 1:** Two Scarab Finger rings found on the 3^rd^ finger of the left hand of mummy (CIT 8). Currently displayed at The Metropolitan Museum of Arts, New York

**Figure 1A:** Red jasper scarab on a thin silver ring. The bottom of the scarab inscribed ‘Amenhotep Ra’. This was the inner ring on the finger

**Figure 1B**: Green jasper scarab with gold swivel mounting on gold ring. The bottom of the scarab shows sm3 t3wy symbolises the union of the two lands or kingdoms of Upper and Egypt, it portrays the lungs and windpipe being connected by papyrus on one side and lotus on the other.


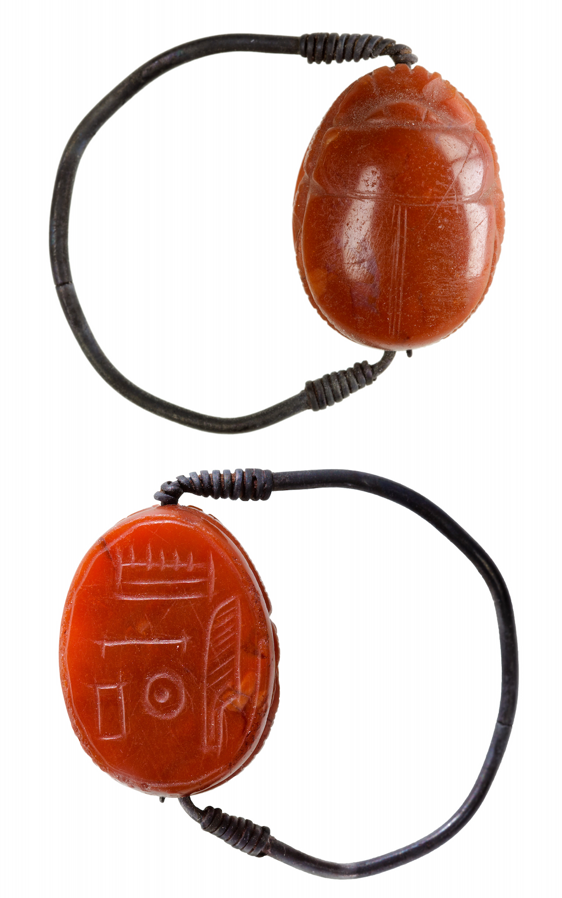

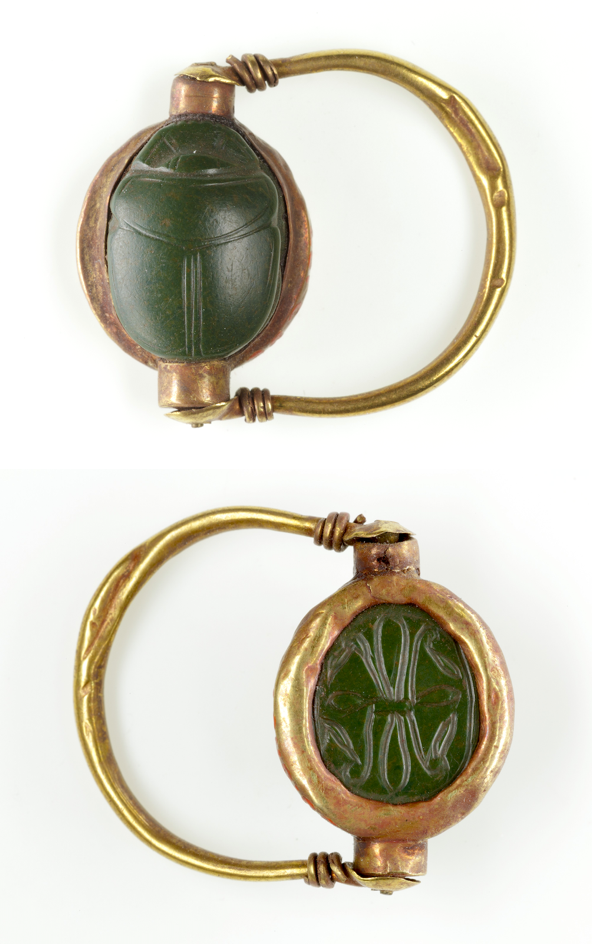


**Supplementary Table 1:** Computed Tomography (CT) scanning parameters used in the study

| Mummy region | Slice thickness in millimeter (mm) | Field of View (FOV) | Reconstruction kernel |
| --- | --- | --- | --- |
| Head | 0.6 | 320 | B20 (soft tissues)  B60 (bones) |
| Teeth | 0.6 | 320 | H70 (high resolution) |
| Chest-abdomen | 0.6 | 320 | B20s (soft tissues)  B60s (bones) |
| Pelvis | 0.6 | 320 | B20 (soft tissues)  B60 (bones) |
| Lower limb | 0.6 | 320 | B20 (soft tissues)  B60 (bones) |
| Body | 1.25 | 450-500 | B20 |

Note: CT scanner: Siemens Medical Solutions, Florsheim, Germany
